# Optical Conductivity and Photo‐Induced Polaronic Formation in Co_2_MnGa Topological Semimetal

**DOI:** 10.1002/advs.202400247

**Published:** 2024-09-09

**Authors:** Luca Tomarchio, Salvatore Macis, Sen Mou, Lorenzo Mosesso, Anastasios Markou, Edouard Lesne, Claudia Felser, Stefano Lupi

**Affiliations:** ^1^ Department of Physics Sapienza University Piazzale Aldo Moro 5 00185 Rome Italy; ^2^ INFN section of Rome P.Le Aldo Moro, 2 00185 Rome Italy; ^3^ Physics Department University of Ioannina 45110 Ioannina Greece; ^4^ Max Planck Institute for Chemical Physics of Solids Nöthnitzer Str. 40 01187 Dresden Germany

**Keywords:** photoconductivity, polarons, THz, topological semimetal

## Abstract

Topological materials occupy an important place in the quantum materials family due to their peculiar low‐energy electrodynamics, hosting emergent magneto‐electrical, and nonlinear optical responses. This manuscript reports on the optical responses for the magnetic topological nodal semimetal Co_2_MnGa, studied in a thin film geometry at various thicknesses. The thickness‐dependent optical conductivity is investigated, observing a substantial dependence of the electronic band structure on thickness. Additionally, details on the ultrafast response of the low energy excitations in the terahertz frequency are reported by employing optical pump‐terahertz probe (OPTP) spectroscopy. In particular, the photocarrier dynamics of Co_2_MnGa thin films is studied at varying pump fluence, pump wavelength, and film thickness, observing a negative THz photoconductivity which is assigned to a dynamical formation of large polarons in the material.

## Graphical abstract

Topological materials host peculiar low‐energy electrodynamics which can be tuned via different means. Using optical pump‐THz probe spectroscopy, this study reveals the dynamical formation of polaronic quasiparticles and their contribution to the THz conductivity after optical illumination in the topological semimetal Co_2_MnGa.

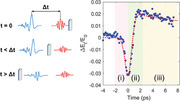

## 1 Introduction

The breaking of time‐reversal symmetry through the presence of intrinsic magnetic effects further increases the interest in topological materials, giving rise to novel topological phases of matter, like the magnetic topological insulators,^[^ ^1^, ^2^, ^3^, ^4^ ^]^ semimetals,^[^ ^5^, ^6^, ^7^ ^]^ and axion insulators.^[^ ^8^ ^]^ Quantum anomalous Hall effects (AHE),^[^ ^9^, ^10^, ^11^ ^]^ with the appearance of chiral dissipationless edge channels, half‐quantized surface anomalous Hall conductivity^[^ ^8^ ^]^ and nonlinear optical effects like multiple harmonic generations (MHG)^[^ ^12^, ^13^, ^14^ ^]^ and circular photogalvanic effect (CPGE)^[^ ^15^, ^16^ ^]^ are just a few examples of the intriguing electrodynamics in these topological phases.

**Figure 1:**
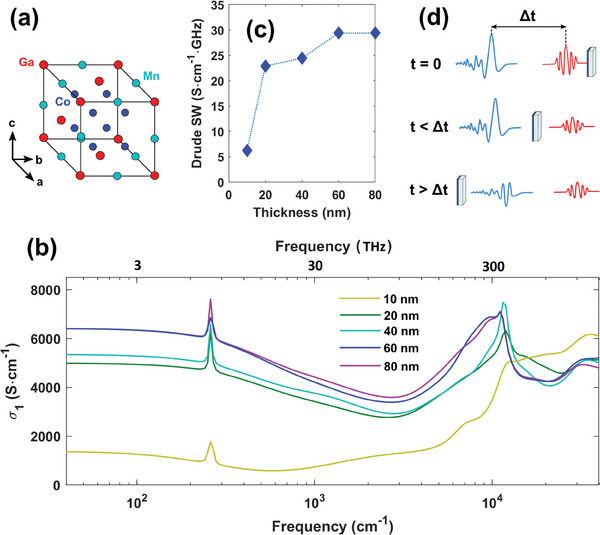
Linear optical response of Co_2_MnGa thin films. a) Cubic unit cell of a Co_2_MnGa crystal. The structure is understood as being the result of four interpenetrating face‐centered‐cubic (fcc) lattices (Heusler alloy structure). The arrows indicate the crystal axes. b) Real part of the optical conductivity of CMG films with different thicknesses, from THz to UV. c) Drude spectral weight as a function of thickness, showing the convergence to a bulk behavior. d) Optical pump‐THz probe spectroscopy measurements. The THz signal (light blue) is transmitted across the sample at a time Δ*t* after the arrival of the optical pump (red). Varying Δ*t* permits sampling the out‐of‐equilibrium dynamics of the low‐energy electrons.

The full‐Heusler alloy Co_2_MnGa (CMG) has been theoretically predicted^[^ ^17^, ^18^ ^]^ and experimentally proven^[^ ^19^ ^]^ to be a magnetic topological nodal line semimetal with valence and conduction bands crossing along arbitrarily intertwined nodes.^[^ ^20^, ^21^ ^]^ Moreover, CMG shows a Curie temperature higher than 600 K, crystallizing in the cubic Cu_2_MnAl‐type structure with space group $\mathrm{Fm}\bar{3}m$ ^[^ ^22^, ^23^ ^]^ (see **Figure** **1**).

Despite the promising properties of CMG, not much is known about its electronic properties and the corresponding optical behavior.^[^ ^24^, ^25^, ^26^, ^27^, ^28^ ^]^ In this framework, optical linear spectroscopy and optical pump‐terahertz probe (OPTP) spectroscopy are key tools for the characterization of the electronic features of these semimetals. Photoconductive responses have been used to study the photocarrier scattering mechanism governed by the unique band structure in Dirac semimetals^[^ ^29^, ^30^, ^31^, ^32^ ^]^ and graphene,^[^ ^33^, ^34^ ^]^ highlighting the dynamical interaction between charge, lattice, and even spin degrees of freedom. Understanding the sub‐ps dynamics of topological materials, like CMG, allows shedding light on the interaction between the electronic, lattice, and magnetic degrees of freedom in view of their applications in electro‐optics devices.

In this work, the optical properties of CMG thin films with different thicknesses, ranging from 10–80 nm, and grown on MgO (100) substrates are studied. The frequency‐dependent broadband optical response of CMG highlights a subtle dependence of the optical conductivity on the film thickness, mainly related to strains induced by the underlying substrate. Moreover, time‐resolved optical‐pump/Terahertz‐probe spectroscopy at different pumping wavelengths, from 800 to 6000 nm, and various fluences are employed to elucidate the photocarrier dynamics. This study reveals an absorption enhancement of the THz radiation due to the photoinduced mobile carriers. This enhancement is followed by a transmittance increase on a sub‐picosecond timescale. This effect can be associated to a reduction of the photoinduced carrier's mobility due to the formation of dynamical polarons in Co_2_MnGa.

## 2 Results

High‐quality epitaxial thin films of Co_2_MnGa were grown in a BESTEC UHV magnetron sputtering system on single crystal MgO(001) substrates and capped with 3 nm Al, which is naturally oxidized and protects the films. Details of the film growth are provided in the Experimental Section. The cubic crystal structure of CMG is shown in Figure 1a. The arrows indicate the a, b, and c crystal axes. In this work, five films of different thicknesses (10, 20, 40, 60, and 80 nm) have been investigated.

### 2.1 Optical Spectroscopy Results

Reflectance (R) and transmittance (T) have been measured at room temperature in a broad spectral range from THz (40 cm^−1^) to UV (50 000 cm^−1^) (≈2.5 meV – 6.2 eV). The spectroscopy setup is discussed in the Experimental Section. In Figure 1b the real part of the films' conductivity is reported across the broadband spectrum, as extracted through a Kramers–Kronig consistent fitting process of the film response in a trilayer air/film/substrate system.^[^ ^35^ ^]^ The multi‐layer stacking model description can be consulted in the Supporting Information (SI) with the imaginary part of the conductivity (Figure S1, Supporting Information). The optical conductivity is dominated by a Drude term up to the infrared range, whose spectral weight (SW) is shown in Figure 1c as a function of thickness. This quantity follows a first rapid increase from 10 to 20 nm, smoothly saturating at the maximum thickness here measured (80 nm). The sharp peak at ≈260 cm^−1^ is associated to a CMG phonon (*E*_*u*_).^[^ ^36^ ^]^ At higher frequencies, several broad features related to electronic interband transitions can be observed, well separated from the Drude term by a pronounced minimum at 3000 cm^−1^. In particular, for the thicknesses 20, 40, 60, and 80 nm, a main peak can be observed ≈10 000 cm^−1^ (1.3 eV), followed by another broad absorption at≈16 000 cm^−1^ (2 eV). These structures have been already observed in a CMG polycrystalline material and are well reproduced by a DFT+U calculation with *U* = 5.4 eV.^[^ ^27^ ^]^ This value of *U* suggests the presence of strong electronic correlations in Co_2_MnGa nodal semimetal. The 10 nm spectrum, instead, shows a notable difference with respect to the thicker films (see Figure 1b). In particular, the minimum ≈3000 cm^−1^ observed for the other thicknesses is now substituted by a broad absorption extending down to the mid‐IR. Additionally, the prominent peak at nearly 10 000 cm^−1^ is strongly reduced and partially shifted at a higher frequency. The shift of the electronic transitions with thickness can be associated to the strains induced onto the film by the MgO substrate as suggested in ref. [37]. Moreover, a similar trend in thickness has been observed with THz emission spectroscopy^[^ ^38^ ^]^ and magneto‐optical measurements,^[^ ^37^, ^39^ ^]^ confirming the role of substrate‐induced strain in the topological properties of Co_2_MnGa.

### 2.2 Optical Pump‐THz Probe Results

To study the temporal dynamics of Co_2_MnGa topological charge carriers over a broad time span, from sub‐ps to nanoseconds, different optical pumps from 800 to 6000 nm were used. The photoinduced current is then probed by a THz pulse in a transmission configuration (see Figure 1d for the optical scheme). More specifically, the transmitted THz electric field change Δ*E*_*t*_ = *E*_*t*_ − *E*_0_ is measured as a function of the pump‐probe delay time Δ*t* between the optical and THz signal, where *E*_*t*_ and *E*_0_ denote the peak value of the THz electric field at a delay time *t* and without the a pump, respectively. Due to the low transmission of the 80 nm film, only the 20, 40, and 60 nm samples have been studied. In the thin film approximation, the photoinduced relative transmission change Δ*E*_*t*_/*E*_0_ is proportional to the negative THz photoconductivity (NPC) −Δσ_1_/σ_1_, where σ_1_ is the real part of the optical conductivity.

**Figure 2:**
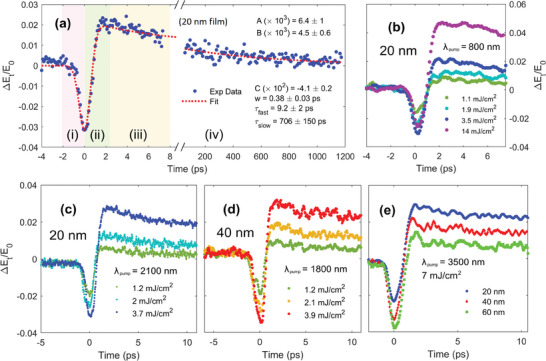
OPTP spectroscopy of Co_2_MnGa thin films. a) Short and long‐time trend of the photoinduced THz peak transmission change for a 20 nm CMG film pumped with a 3.5 mJ cm^−2^ pump with 800 nm central wavelength. The colored boxes highlight the four‐steps process of transient excitation (i, ii) and relaxation to equilibrium (iii, iv). The red dotted line identified the best‐fitting process using Equation (1). b,c) OPTP measurements at two different pump wavelengths for the 20 nm thick sample. Different fluences have been measured at each pumping wavelength. e) OPTP result for a 40 nm film pumped by a 1800 nm pulse at different fluences. f) OPTP measurements for three CMG films of 20, 40, and 60 nm, using a 3500 nm pulse and 7 mJ cm^−2^ fluence.

**Figure** **2**a shows Δ*E*_*t*_/*E*_0_ for the 20 nm film at a fixed pumping wavelength of 800 nm and for an optical fluence of 3.5 mJ cm^−2^. The time axis is separated into two parts to highlight the temporal behavior in a short and long time window. Four stages with distinct time scales for the photoinduced response can be highlighted: i) a first increase in photoconductivity (decrease in THz amplitude transmission); ii) a second rapid (sub‐picosecond) transition to a positive PC; iii) a fast (few picoseconds) decay process from the maximum PC value to a positive plateau; iv) a slow (hundreds of picoseconds) recovery process to equilibrium. Figure 2b,c show the photoinduced THz transmission for the 20 nm film at 800 nm (b) and 2100 nm (c) pumping wavelengths and for different pumping fluences. The same results are shown in Figure 2d for the 40 nm sample using an 1800 nm pump. Other results for both the 20 and 40 nm films are shown in Figure S2 (Supporting Information). Figure 2e shows instead the comparison between the pump‐probe effect at 3500 nm for the three films: 20, 40, and 60 nm. Similar comparisons for other wavelengths are shown in Figures S3 and S4 (Supporting Information). These results suggest that the four‐step process is valid for all the pumping wavelengths, from visible to mid‐infrared, and film thicknesses.

## 3 Discussion

The change in the THz conductivity Δσ = Δ(*n*μ)*e*, where *e* is the elementary electric charge, can be associated with the change in the number of carriers (Δ*n*) and/or on their mobility (Δμ) under photoexcitation. Thus, the time evolution of terahertz transmission after photoexcitation is governed by Δ*n* and/or Δμ. Due to the high carrier density of the metallic films (≈10^22^ cm^−3^),^[^ ^25^ ^]^ the former contribution is small even at the highest fluence employed here. Indeed, considering the absorption coefficient of the 40 nm film (similar results can be obtained for the other thicknesses), one can estimate Δ*n*(λ, *d* = 40 nm) for a 3.5 mJ cm^−2^ at different wavelengths λ as: Δ*n*(800 nm) ≈4 × 10^19^ cm^−3^, Δ*n*(1300 nm) ≈3.5 × 10^19^ cm^−3^, and Δ*n*(2100 nm) ≈3 × 10^19^ cm^−3^. These values are nearly three orders of magnitude lower than the equilibrium charge density and comparable across the whole film's thickness range. Therefore, in region (i) (see Figure 2a) the photoexcitation mainly results in a broadening of the Fermi distribution of hot carriers due to electron‐electron thermalization over a wide energy range.^[^ ^40^ ^]^ This leads to an increase of free carriers THz absorption due to intraband transitions having larger momentum and energy conservation phase space.^[^ ^40^ ^]^

In region (ii), the positive value of the THz transmitted field can be instead associated with a reduction in the charge‐carrier mobility, extending up to a nanosecond scale (regions iii and iv). Similar effects have been observed in materials such as graphene,^[^ ^41^, ^42^ ^]^ transition metal dichalcogenides (TMDs),^[^ ^43^ ^]^ Dirac semimetals,^[^ ^40^ ^]^ and perovskytes.^[^ ^44^, ^45^ ^]^ In the last three references the positive value of the THz transmittance (the negative photoconductivity) has been explained in terms of a dynamical polaronic formation.

To analyze the whole pump‐probe data, the transient THz field transmission in Figure 2a can be fitted through a sum of a Gaussian term describing the fast dynamics in (i) and two mono‐exponential functions^[^ ^40^, ^46^ ^]^

(1) $$\begin{array}{cc} \frac{\Delta E_{t}}{E_{0}} & =Ce^{-t^{2}/4w^{2}}+Ae^{-\frac{t}{\tau_{fast}}}\left[1-\mathrm{erf}\left(\frac{w}{\tau_{fast}}-\frac{t}{2w}\right)\right]\\ & \;+Be^{-\frac{t}{\tau_{slow}}}\left[1-\mathrm{erf}\left(\frac{w}{\tau_{slow}}-\frac{t}{2w}\right)\right] \end{array}$$

where τ_*fast*_ and τ_*slow*_ are the fitted relaxation times for processes (iii) and (iv), respectively. The rise time from positive to negative PC of process (ii) is instead parametrized by the parameter *w* and modeled through an activation function 1 − erf(·), where erf is the error function. The fitting is highlighted in Figure 2a as red dotted curves, from which the time constants for the four processes are extracted: *w* = 0.38 ± 0.03 ps, τ_*fast*_ = 9.2 ± 2 ps, and τ_*slow*_ = 0.71 ± 0.15 ns (all the fit parameters are shown in Figure 2a).

The positive value in the THz transmitted field after the photoexcitation process (region i) can be associated with the formation of polaronic quasiparticles with reduced mobility (region ii). Other possible mechanism for the reduced mobility will be discussed and discarded later in this section. Region (iii) is instead related to the polarons cooling process resulting in an increase of their conductivity. Finally, region (iv) is related to the decay of polarons toward the equilibrium state. In particular, upon the photoexcitation process (region i), the photogenerated hot carriers strongly interact with the CMG lattice vibrations. The electron‐phonon (e‐ph) coupling yields the cooling of hot carriers and the consequent distortion of the lattice around the carriers gives rise to polaronic charges with reduced carrier mobility, which is the origin of the terahertz negative photoconductivity. The change in mobility in the region (ii) as a consequence of the polaronic formation takes place on a sub‐picosecond scale, with a time constant of the order of 400 fs for all wavelengths and fluences. Similar time constants have been found for the polaronic dynamical formation in different materials ranging from polar lead‐iodide perovskites^[^ ^44^, ^45^ ^]^ to type‐II high‐conductivity PtTe_2_ Dirac semimetals.^[^ ^40^, ^47^ ^]^ The changes in magnitude for the pump‐probe traces at different film thicknesses (see Figure 2e; Figure S5, Supporting Information) are addressed in terms of both a stronger electron screening in the 40/60 nm films (see Figure 1b), hindering the coupling with the lattice, and the enhanced strain effects from the substrate in the 20 nm film, as discussed in the Supporting Information.

The polaron cooling process is subdivided into two steps, ascribed to regions (iii and iv). The former identifies a fast cooling process with a consequent increase in the polaron mobility. Indeed, the relaxation time values obtained from Equation (1) (τ_*fast*_) fall on a temporal scale of a few picoseconds. Similar time constants were found in other magnetic thin films,^[^ ^28^, ^46^, ^48^ ^]^ and could be associated with a fast electron‐lattice or electron‐spin‐lattice thermalization that takes place after the fast polaron formation. Finally, the slow relaxation process (iv), of the order of hundreds of picoseconds, suggests a long lifetime for the new less‐mobile carriers, which can be related to the slow bottleneck decay of the polaronic quasiparticles due to the low thermal conductivity of CMG.^[^ ^49^ ^]^

**Figure 3:**
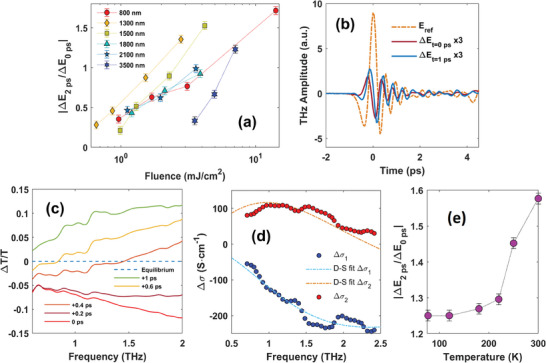
Polaron excitation in Co_2_MnGa thin films. a) Ratio between the transmitted electric field change Δ*E*_*t*_ after (2 ps) and before the polaron formation (0 ps) as a function of fluence for different pumping wavelengths. b) Photoconductive changes induced in the THz electric field transmitted by the 20 nm CMG film after an optical pump at 1300 nm and with a 0.71 mJ cm^−2^ fluence. *E*_*ref*_ identifies the original signal transmitted without the pump application. c) THz transmission change in the 20 nm film for different time delays between the optical pump at 1300 nm and the THz probe. A transparency effect is induced after the polaronic formation due to the lower mobility of electrons. The effect is stronger at higher frequencies. d) Real (blue dots) and imaginary (red dots) part of the THz photoconductivity (at Δ*t* = 2 ps) for the 20 nm film at 1300 nm pump wavelength. The dashed curves describe a Drude–Smith fit whose parameters are reported in the main text. e) Ratio between the transmitted electric field change Δ*E*_*t*_ after (2 ps) and before the polaron formation (0 ps) as a function of temperature for a 20 nm thick film.

This polaronic scenario is fully consistent with OPTP measurements at different fluences and wavelengths. Indeed, one can notice an enhancement of the NPC signal for a pump fluence increase (see Figure 2b–d). A higher pump fluence induces a higher charge‐carrier temperature, therefore more phonons are excited during the hot electron cooling process (region i) via the e‐ph coupling. As a result, more charge carriers are “trapped” through the electron‐phonon interaction and the NPC signal under higher pump fluence becomes more pronounced. **Figure** **3**a reports on the fluence dependence for different wavelengths by plotting the ratio Δ*E*_2 *ps*_/*E*_0 *ps*_ for the 20 nm film. Here, Δ*E*_2 *ps*_ and Δ*E*_0 *ps*_ are the THz electric fields after (2 ps) and before (0 ps) the polaron formation. On the semi‐log scale, a nearly linear dependence on fluence can be observed for all wavelengths, from 800 nm to 3500 nm. However, higher magnitudes are found when pumping in the NIR, near 1300 nm. This effect can be a consequence of the strong interband transitions at the Γ point for CMG. Here, the electrons are pumped from below the Fermi level to the parabolic conduction band, as predicted by DFT calculations,^[^ ^49^ ^]^ inducing a peak light absorbance close to the 1300 nm pump. Figure S8 (Supporting Information) highlights these resonant transitions on the band structure and conductivity plot for CMG. The independence of the probe signal independence from the pump wavelength (down to the mid‐infrared region) rules‐out the mass renormalization mechanism in the cooling process. Moreover, the possible contribution of trions formation can be also excluded due to the strong electronic screening of CMG.

To further address the polaronic behavior of photoexcited charge carriers in CMG, measurements were carried out by sampling the THz spectrum of the 20 nm sample between 0.5 and 2 THz at different pump delay times for the 1300 nm wavelength. Figure 3b shows the photoinduced change (at 0 and 1 ps) of the THz electric field Δ*E*(*t*_*s*_, *t*) = *E*(*t*_*s*_, *t*) − *E*_ref_(*t*_*s*_) transmitted by the 20 nm film for a 1300 nm pump wavelength and a 0.71 mJ cm^−2^ pump fluence. Here, *E*_ref_ identifies the signal transmitted without pump, while *t*_*s*_ is the electro‐optical probing time of the THz scan. In Figure 3c we plot the relative transmittance Δ*T*/*T* as a function of frequency for a sequence of pump delay times. At early times Δ*T*/*T* shows negative values indicating an absorption enhancement due to the hot charge‐carrier formation. Δ*T*/*T* progressively changes sign at longer times in agreement with the NPC process. The frequency dependence of the THz transmittance is compatible with a polaronic formation as discussed below, while disproving the idea of a simple thermal effect contributing to the reduced mobility.

On general grounds,^[^ ^50^ ^]^ polaronic quasiparticles can be roughly classified into two different families: large and small polarons. Large (small) polarons correspond to a lattice distortion much larger (comparable) to the unit cell size and possess a coherent Drude (thermally induced hopping) motion.^[^ ^51^, ^52^ ^]^ To identify the polaron nature in Co_2_MnGa, we study the real (blue points) and imaginary (red points) THz conductivity as extracted from the time‐domain data at Δ*t* = 2 ps for the 20 nm film at 1300 nm pump wavelength. The experimental data, shown in Figure 3d, does not follow a Drude behavior and can be fitted to a Drude–Smith (D‐S) model, described by the Equation σ(ω) = σ_0_/(1 − *i*ω/γ) × (1 + *c*/(1 − *i*ω/γ)), where *c* = −1, γ = 2.4 cm^−1^, and σ_0_ = −465 S·cm^−1^ from the best fit procedure. The best fit curves using this model are shown in Figure 3d as dashed lines. A D‐S conductivity has been observed in many large‐polaron systems,^[^ ^50^ ^]^ supporting the dynamical formation of large polaron in Co_2_MnGa nodal semimetal. A second proof supporting this picture comes from the temperature dependence of the pump‐probe response of the 20 nm film, measured from 300 to 77 K. By lowering the temperature, the ratio Δ*E*_2*ps*_/*E*_0*ps*_ is reduced, highlighting how the polaron mobility increases at lower temperatures, as expected for large polarons. This ratio is shown in Figure 3e for the 20 nm film and a 1300 nm pump wavelength with a 2 mJ cm^−2^ fluence.

## 4 Conclusion

In this study, we elucidated the optical properties of thin films of the novel magnetic topological nodal line semimetal Co_2_MnGa across varying thicknesses. Employing broadband linear spectroscopy, we conducted comprehensive conductivity characterizations spanning frequencies from THz (40 cm^−1^) to UV (50 000 cm^−1^). Our findings reveal a thickness‐dependent tuning of the low‐energy electrodynamics, where the thinnest film (10 nm) exhibits an optical conductivity strongly influenced by substrate strain, transitioning to a more “bulk” response at increased thicknesses. Furthermore, we delved into the out‐of‐equilibrium low energy response through optical pump‐terahertz probe (OPTP) measurements. These experiments unveiled the formation of large polaronic quasiparticles, allowing us to track their temporal dynamics in relation to pumping wavelength and fluence. The results indicate a robust coupling between electrons and the lattice, with a nuanced dependence on film thickness and pumping wavelength. This work provides crucial insights into the photocarrier dynamics of the novel nodal line semimetal CMG. The precise understanding of low‐energy electrodynamics, especially regarding the formation of large polaronic quasiparticles, holds significant implications for the advancement of ultrafast, topological‐based optoelectronic, and thermoelectric devices. Our research contributes to the foundational knowledge necessary for developing innovative technologies in this burgeoning field.

## 5 Experimental Section

### Thin Films Growth

Thin films of Co_2_MnGa with thicknesses of 10, 20, 40, 60, and 80 nm were grown epitaxially, on (001)‐oriented MgO single‐crystal substrates, using a BESTEC ultra‐high vacuum magnetron sputtering system. Prior to the deposition, the chamber was evacuated to a base pressure of less than 8 × 10^−9^ mbar, while the process gas (Ar 5 N) pressure was set to 3 × 10^−3^ mbar. The films were grown using an equiatomic compound Mn_50_Ga_50_ and high‐purity elemental metals Co and Mn sources in a confocal geometry, 20, 34, and 6 W DC power applied, respectively. The target‐to‐substrate distance was fixed at 20 cm, and the substrate was rotated with a speed of 20 rpm during deposition to ensure a homogeneous growth. The films were grown at 600 °C, followed by in situ post‐annealing at the same temperature for 30 min to improve the crystallinity of the films. To avoid oxidation of the films, an amorphous Si layer of 3 nm was deposited in situ at room temperature using a Si (5.08 cm) source and applying 60 W RF power.

### Optical Characterization

Transmission and Reflectance measurements at room temperature were taken through a Vertex 70v FTIR broadband interferometer, covering the spectral range from THz (40 cm^−1^) to MIR (7000 cm^−1^), and through a Jasco V‐770 spectrometer, extending the spectral data from 5000 cm^−1^ up to the UV region (50 000 cm^−1^). Optical pump‐terahertz probe measurements were developed in a transmission geometry through the generation of THz pulses from a ZnTe (110) crystal, whose electric field is detected in a time‐domain experimental configuration through an electro‐optical sampling in a twin ZnTe. All measurements were performed in dry air. The signal covered a range of frequencies going from 0.6 to ≈2.5 THz. It was generated by an 800 nm pulse with a time duration of 35 fs, as obtained by a Ti:sapphire amplifier (Coherent Verdi G‐series), with a repetition rate of 1 kHz. The signals used to pump the films along with the THz were obtained from the 800 nm itself which could be used directly or sent to an optical parametric amplificator (TOPAS Prime from Light Conversion) in series with a difference‐frequency generation (NDFG from Light Conversion) for the generation of wavelengths from 1200 to 6000 nm. A 400 nm pump was also been obtained from the second harmonic generation of the 800 nm signal into a BBO nonlinear crystal. The THz detection was achieved through an electro‐optical scheme based on the Pockels effect induced by the THz radiation itself into a ZnTe crystal. The change in birefringence was probed through an 800 nm pulse overlapping in time and space with the THz pulse in the detection crystal. The polarization change was then measured via a balanced photodiode. The low‐temperature measurements were performed through the use of a He‐cooled JANIS (ST‐100‐FTIR) cryostat.

## Conflict of Interest

The authors declare no conflict of interest.

## Author Contributions

The project was conceptualized and supervised by S.L. and C.F. Samples were synthesized and characterized by A.M and E.L. Reflectance and transmittance measurements were carried out by L.T., S. Macis, and L.M. Optical pump‐THz Probe measurements were carried out by L.T. and S. Mou. The data treatment and analysis were performed by L.T. and L.M. L.T. and S.L. prepared the original draft. All authors reviewed and edited the manuscript.

All authors have read and agreed to the published version of the manuscript.

## Supporting information

Supporting Information

## Data Availability

The data that support the findings of this study are available from the corresponding author upon reasonable request.
