# Supplementary material for: Optical Conductivity and Photo‐Induced Polaronic Formation in Co2MnGa Topological Semimetal
Source: Adv Sci (Weinh). 2024 Sep 9;11(41):2400247. doi: 10.1002/advs.202400247 (PMC11538699; doi:10.1002/advs.202400247)
Supplement: Supplementary file 1 — Supporting Information [file ADVS-11-2400247-s001.pdf]

ADVANCED SCIENCE
Open Access

Supporting Information

for *Adv. Sci.*, DOI 10.1002/advs.202400247

Optical Conductivity and Photo-Induced Polaronic Formation in Co_2MnGa Topological Semimetal

*Luca Tomarchio, Salvatore Macis, Sen Mou, Lorenzo Mosesso, Anastasios Markou, Edouard Lesne, Claudia Felser and Stefano Lupi**

Supplementary Information: Optical Conductivity and Photo-induced Polaronic Formation in Co_2MnGa Topological Semimetal

Luca Tomarchio,[1,2] Salvatore Macis,[1,2] Sen Mou,[2] Lorenzo Mosesso,[1]
Anastasios Markou,[3] Edouard Lesne,[3] Claudia Felser,[3] and Stefano Lupi[1,2,*]
[1]*Department of Physics, Sapienza University, Piazzale Aldo Moro 5, 00185, Rome, Italy.*
[2]*INFN section of Rome, P.Le Aldo Moro, 2, 00185 Rome, Italy.*
[3]*Max Planck Institute for Chemical Physics of Solids, Nöthnitzer Str. 40, 01187 Dresden, Germany*

I. MULTI-LAYER STACKING MODEL

The reflectivity ρ of a single film on a substrate can be calculated explicitly in terms of the frequency-dependent complex refractive index $\tilde{n}$, the angle of incidence ϕ, the film thickness d_1 and the frequency of the incoming light ν (in wavenumbers)[1]. For a stacking of multiple layers at normal incidence, such as the air/film/Al_2O_3 trilayer in Fig. **S1**c, respectively indexed as 0/1/2, the complex reflectivity equation is given by

$$\rho = \frac{r_{0,1} + r_{1,2}\exp(-2i\delta_1)}{1 + r_{0,1}r_{1,2}\exp(-2i\delta_1)}, \qquad \delta_1 = 2\pi\tilde{n}_1\nu d_1 \tag{1}$$

Where $r_{m,n}$ is the Fresnel coefficient describing the reflectivity from an interface. The intensity reflectance is obtained by taking the absolute value of the reflectivity ρ.

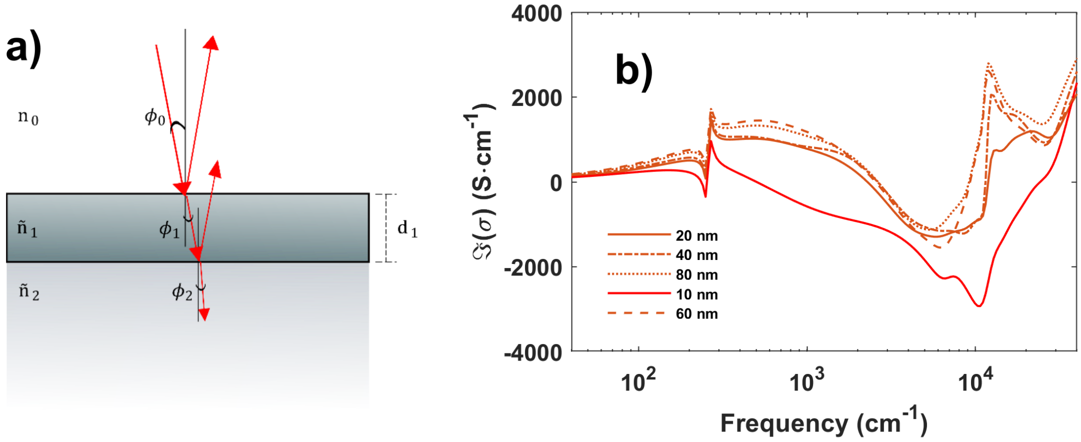

Figure S1: | **Multi-layer stacking model and linear optical response of Co_2MnGa thin films. a)** Schematic representation of the multiple reflections occurring in a single film ($\tilde{n}_1$, d_1) deposited on a substrate ($\tilde{n}_2$) and in contact with air (n_0). **b)** Imaginary part of the optical conductivity of CMG films with different thicknesses, from THz to UV.

II. OPTICAL PUMP-THZ PROBE (OPTP) SPECTROSCOPY: NIR PUMP

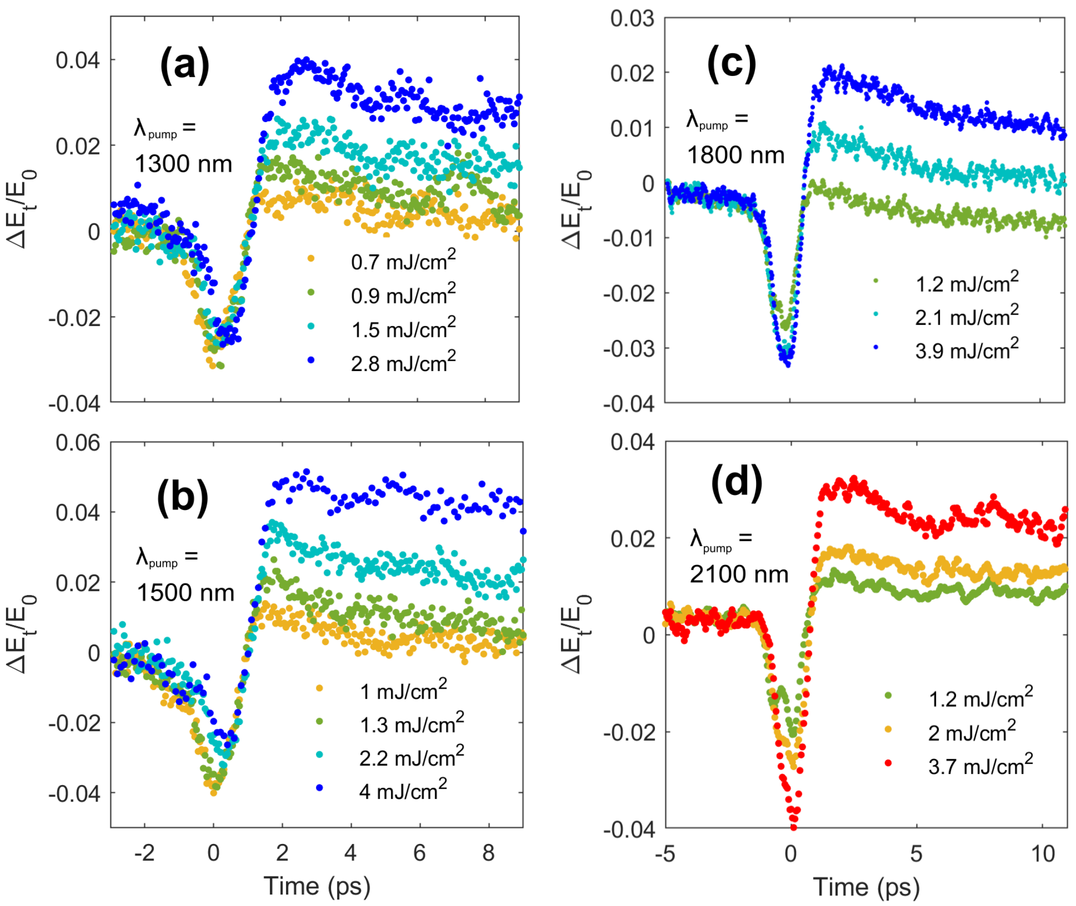

Figure S2: **| NIR pump-THz probe (OPTP) of CMG films. a-d)** OPTP spectra in time for different pumping wavelengths, fluences, and thicknesses.

III. OPTP: 400 NM

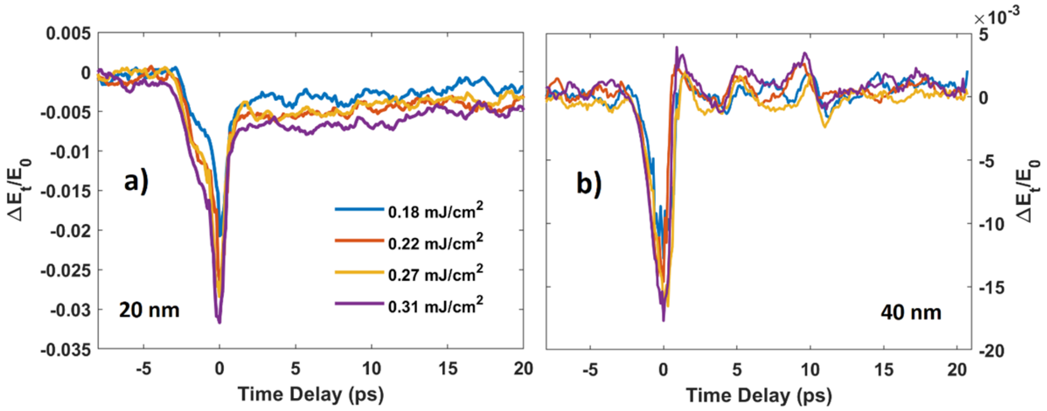

Figure S3: **| OPTP results for a pumping wavelength of 400 nm. a)-b)** Photoinduced variation in the THz field transmitted after the pump at 400 nm for the 20 nm and 40 nm films, respectively

IV. OPTP SPECTROSCOPY: MIR PUMP

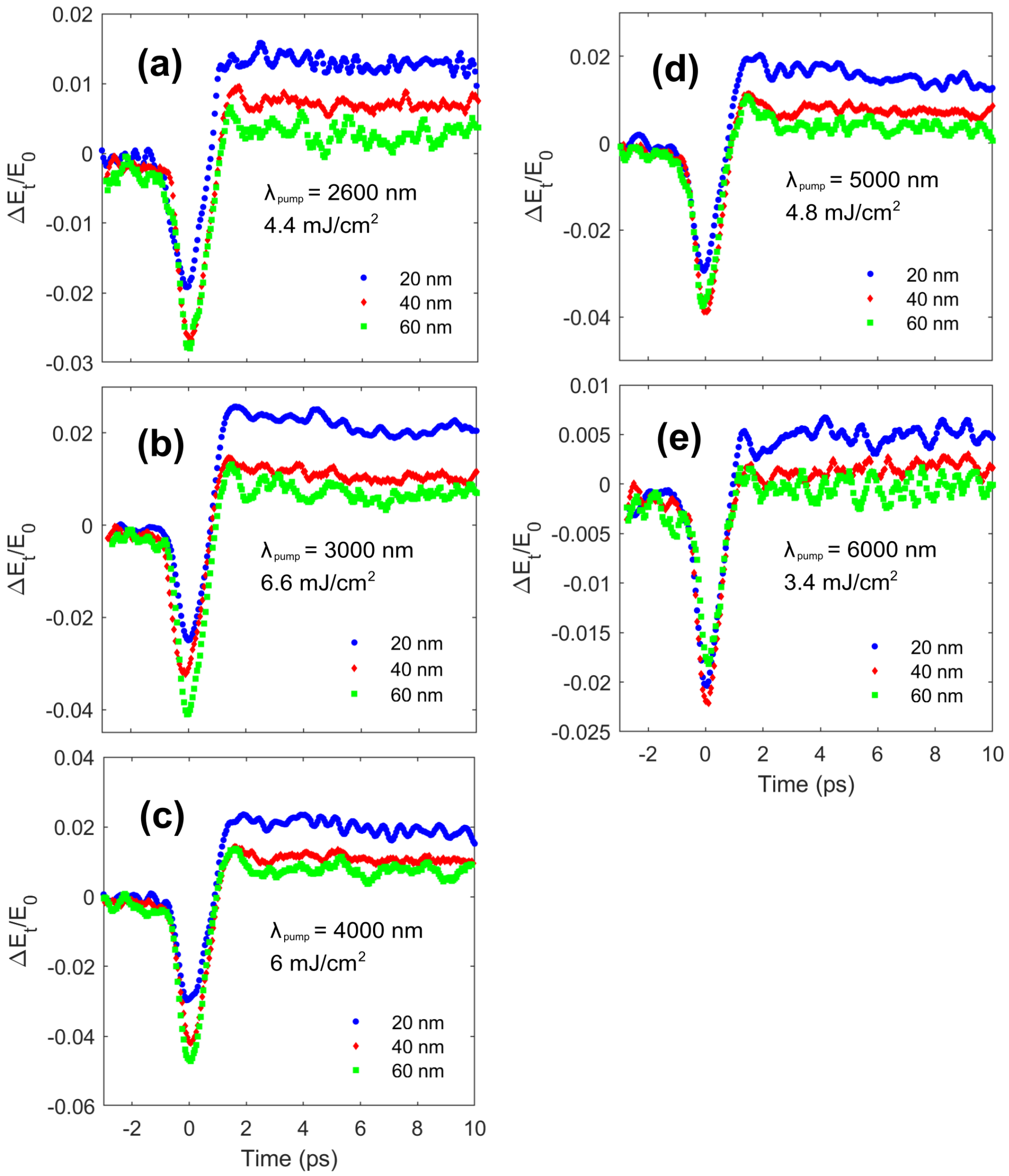

Figure S4: | **MIR pump-THz probe (OPTP) of CMG films. a-e)** OPTP spectra in time for different pumping wavelengths, fluences, and thicknesses.

V. THICKNESS AND PUMP WAVELENGTH DEPENDENCE

To address the thickness dependence, Fig. **S5**a shows a comparison between the polaronic formation in the 20 nm (circle points, blue), 40 nm (diamond points, red), and 60 nm (square points, green) films at different MIR wavelengths. Each point describes the ratio between the transmitted electric field change ΔE_t after (2 ps) and before the polaron formation (0 ps), as a function of the pumping wavelength. The results suggest a stronger e-ph coupling in the thinner film, 20 nm, while the 40 and 60 nm films show a comparable magnitude.

To understand the higher e-ph coupling in the thinner film, two main contributions can be suggested: the stronger electron screening in the 40/60 nm film, hindering the coupling with the lattice, and the enhanced strain effects from the substrate in the 20 nm sample[2–4]. In its long-range approximation, the e-ph coupling constant is proportional to the difference $\propto \epsilon_\infty^{-1} - \epsilon_0^{-1}$[5], where ϵ_∞ and ϵ_0 are the high-frequency (optical) and the static electric constant, respectively. The presence of a polar ($\epsilon_\infty < \epsilon_0$) substrate like MgO can thus induce a stronger e-ph coupling in the thinner 20 nm film.

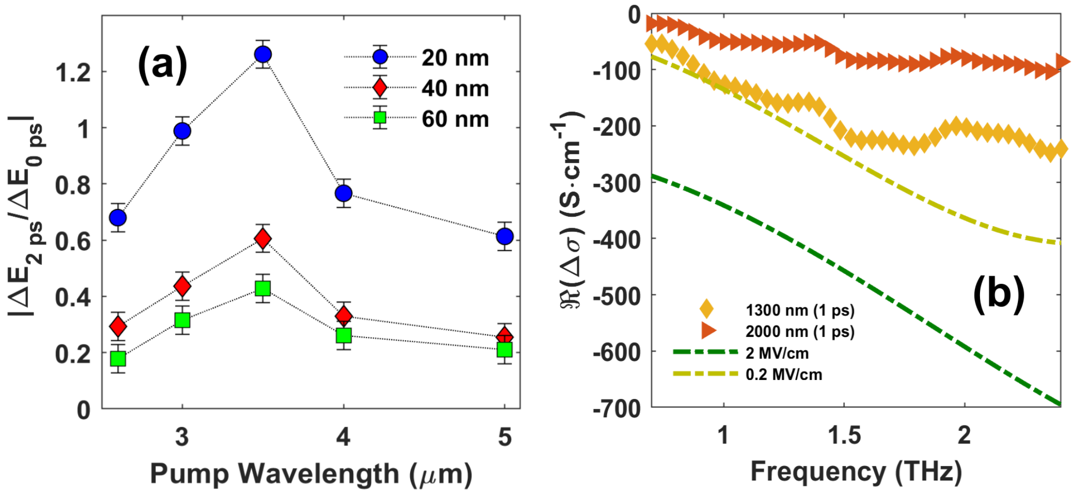

Figure S5: **| Thickness dependence and THz nonlinear photoconductivity. a)** Ratio between the transmitted electric field change ΔE_t after (2 ps) and before the polaron formation (0 ps) as a function of the mid-infrared pumping wavelength for three CMG films of different thicknesses: 20, 40, and 60 nm. The 20 nm sample shows the highest change at all wavelengths. **b)** NPC induced at 1 ps for the 1300 and 2000 nm pumping wavelengths (0.71 mJ/cm^2), compared to the NPC induced by strong THz pulses up to 2 MV/cm.

To highlight this latter effect, nonlinear THz transmission experiments were performed, where a strong THz pulse up to 2 MV/cm is sent across the 20 and 40 nm films, studying the transmission as a function of the THz electric field and frequency. Strong THz pulses were generated through optical rectification of a 1500 nm femtosecond pulse in a DSTMS organic crystal (250 μm thick, 3 mm aperture diameter). The output THz signal has a sub-picosecond duration and spans a broadband range of frequencies (see Fig. **S6**a,b), as measured by an electro-optical detection based on a GaP nonlinear crystal. By focusing the signal to a sub-millimeter diameter, it is possible to reach electric fields from 50 kV/cm up to 2 MV/cm. The transmitted signal at different THz amplitudes has been measured and used to compute the THz transmittance and conductivity variances. Fig. **S7**a shows the changes in transmittance for the 20 nm sample, the only one showing a nonlinear feature. Fig. **S7**b, instead, shows the Drude spectral amplitude change as a function of the THz electric field, saturating at 400 kV/cm. This nonlinear effect coincides with a decrease in the optical conductivity at frequencies comparable with the photoconductivity induced by the formation of polarons, as shown in Fig. **S5**b. This similarity suggests that the strong THz pulses, that produce strains and a polarity mismatch on the thin CMG film lattice, can induce a decrease in mobility similar to the one caused by the optical excitation. This result supports the stronger polaron formation in the thinner film as a consequence of the higher coupling between the film and the substrate polarity, which is known to be an important factor in thinner CMG films[6,7].

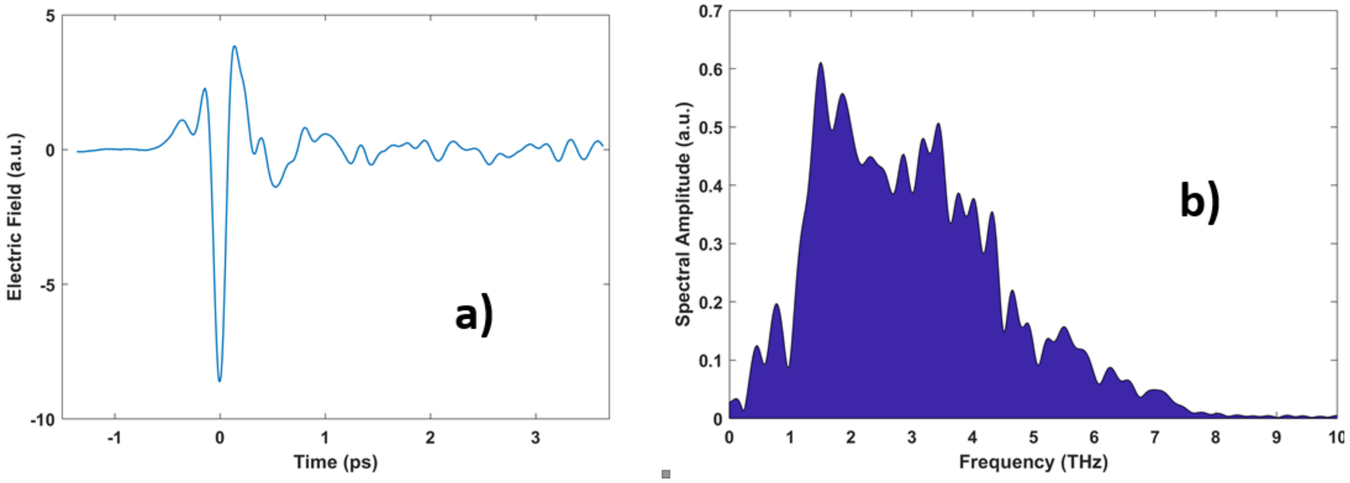

Figure S6: | **THz generation through optical rectification in a DSTMS organic crystal.** **a)** THz electric field as measure by an electro-optical detection in a GaP nonlinear crystal. **b)** Spectral amplitude obtained by fast Fourier transforming the signal in time.

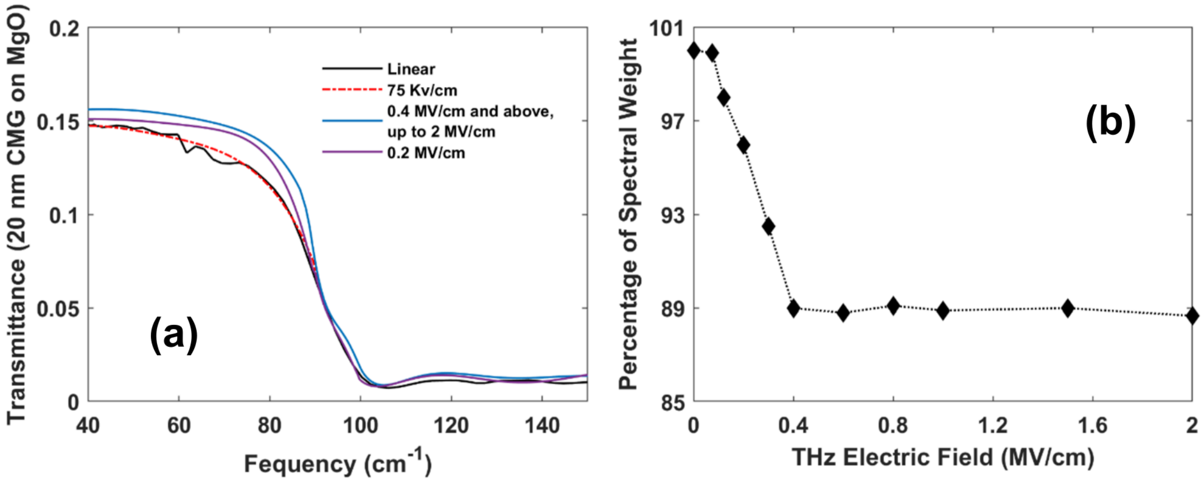

Figure S7: | **Nonlinear transparency of the 20 nm CMG film.** **a)** Transmittance variations at different THz field magnitudes. **b)** Relative variation of the Drude spectral weight for the 20 nm CMG film, as a function of the THz field intensity.

VI. ELECTRONIC TRANSITIONS AFTER PUMPING

The optical pump is absorbed by the electrons of the system that transit to different energy levels in the band structure depending on the pumping wavelength. Fig. **S8**a shows the band structure and the electronic transitions that take place near the Γ point when pumping with $\lambda_pump \simeq 1300$ nm. The same wavelength is shown in Fig. **S8**b over the experimental optical conductivity, along with other pumping wavelengths used in this work for comparison.

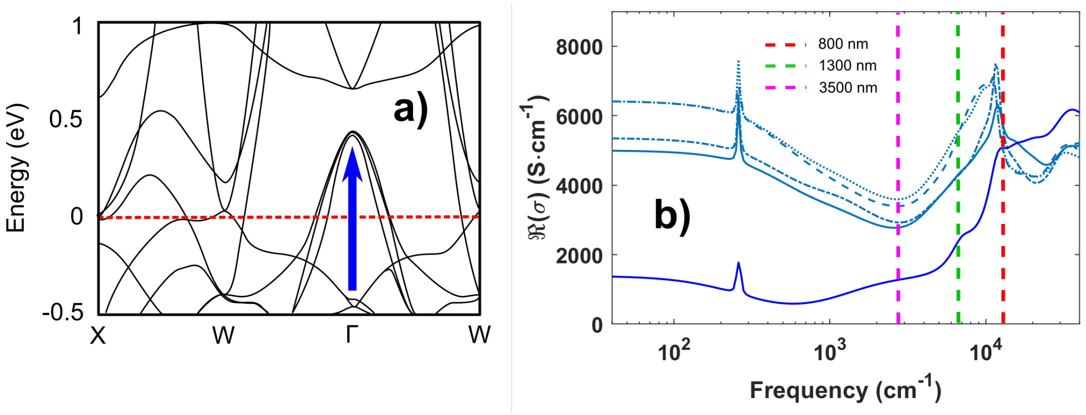

Figure S8: | **Electronic transitions and pump wavelengths. a)** Band structure of CMG (adapted from[8]) with the Fermi level (red line) and the optical transitions at the Γ point for a pump wavelength $\lambda_pump \simeq 1300$ nm. **b)** Overlay of the optical conductivity and three different pump wavelengths.

* Corresponding author: stefano.lupi@roma1.infn.it

[1] O. S. Heavens, Reports on Progress in Physics **23**, 1 (1960).
[2] Z. Zhang, L. You, J. Du, J. Wang, Z. Jin, G. Ma, and Y. Leng, Scientific Reports **8**, 3258 (2018).
[3] B. Guzelturk, T. Winkler, T. W. J. Van De Goor, M. D. Smith, S. A. Bourelle, S. Feldmann, M. Trigo, S. W. Teitelbaum, H.-G. Steinrück, G. A. De La Pena, R. Alonso-Mori, D. Zhu, T. Sato, H. I. Karunadasa, M. F. Toney, F. Deschler, and A. M. Lindenberg, Nature Materials **20**, 618 (2021).
[4] J. Wang, F.-x. Hu, Y.-y. Zhao, Y. Liu, R.-r. Wu, J.-r. Sun, and B.-g. Shen, Applied Physics Letters **106**, 102406 (2015).
[5] C. Franchini, M. Reticcioli, M. Setvin, and U. Diebold, Nature Reviews Materials **6**, 560 (2021).
[6] A. Markou, D. Kriegner, J. Gayles, L. Zhang, Y.-C. Chen, B. Ernst, Y.-H. Lai, W. Schnelle, Y.-H. Chu, Y. Sun, and C. Felser, Physical Review B **100**, 054422 (2019).
[7] P. Swekis, A. S. Sukhanov, Y.-C. Chen, A. Gloskovskii, G. H. Fecher, I. Panagiotopoulos, J. Sichelschmidt, V. Ukleev, A. Devishvili, A. Vorobiev, D. S. Inosov, S. T. B. Goennenwein, C. Felser, and A. Markou, Nanomaterials **11**, 251 (2021).
[8] H. Reichlova, R. Schlitz, S. Beckert, P. Swekis, A. Markou, Y.-C. Chen, D. Kriegner, S. Fabretti, G. Hyeon Park, A. Niemann, S. Sudheendra, A. Thomas, K. Nielsch, C. Felser, and S. T. B. Goennenwein, Applied Physics Letters **113**, 212405 (2018).
